# Radiative Cooling Properties of Portlandite and Tobermorite: Two Cementitious Minerals of Great Relevance in Concrete Science and Technology

**DOI:** 10.1021/acsaom.3c00082

**Published:** 2023-06-23

**Authors:** Jorge S. Dolado, Guido Goracci, Silvia Arrese-Igor, Andrés Ayuela, Angie Torres, Iñigo Liberal, Miguel Beruete, Juan J. Gaitero, Matteo Cagnoni, Federica Cappelluti

**Affiliations:** † Centro de Física de Materiales, CFM (CSIC-UPV/EHU), Paseo Manuel de Lardizabal 5, 20170 Donostia/San Sebastián, Spain; ‡ Donostia International Physics Center (DIPC), Paseo Manuel de Lardizabal 4, 20170 Donostia/San Sebastián, Spain; § Department of Electrical, Electronic and Communications Engineering, Public University of Navarre (UPNA), 31006 Pamplona, Spain; ∥ Institute of Smart Cities (ISC), Public University of Navarre (UPNA), 31006 Pamplona, Spain; ⊥ TECNALIA, Basque Research and Technology Alliance (BRTA), Astondo Bidea, Edificio 700, 48160 Derio, Spain; # Department of Electronics and Telecommunications, Politecnico di Torino, Corso Duca degli Abruzzi 24, Torino 10129, Italy

**Keywords:** Concrete, radiative cooling, cementitious phases, Portlandite, C−S−H gel, Tobermorite, atomistic simulations, homogenization models, scattering, Mie theory

## Abstract

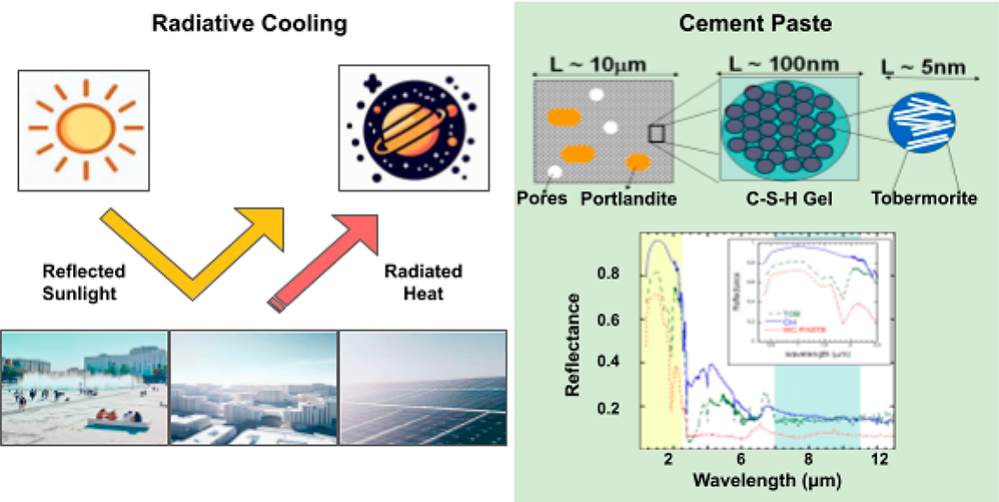

Although concrete and cement-based materials are the most engineered materials employed by mankind, their potential for use in daytime radiative cooling applications has yet to be fully explored. Due to its complex structure, which is composed of multiple phases and textural details, fine-tuning of concrete is impossible without first analyzing its most important ingredients. Here, the radiative cooling properties of Portlandite (Ca(OH)_2_) and Tobermorite (Ca_5_Si_6_O_16_(OH)_2_·4H_2_O) are studied due to their crucial relevance in cement and concrete science and technology. Our findings demonstrate that, in contrast to concrete (which is a strong infrared emitter but a poor sun reflector), both Portlandite and Tobermorite exhibit good radiative cooling capabilities. These results provide solid evidence that, with the correct optimization of composition and porosity, concrete can be transformed into a material suitable for daytime radiative cooling.

## 1 Introduction

Radiative cooling technology^1^ utilizes the atmospheric transparency window (8–13 μm), called the atmospheric window (AW), to passively dissipate heat from the Earth to outer space. All bodies on the Earth are in continuous exchange of energy with the Sun and the atmosphere, with a net balance of power that depends on the incoming solar and atmospheric radiation, the emitted radiation, and nonradiative heat exchanges.

So far, this technology has attracted wide interest from both fundamental and applied sciences, due to its *a priori* high potential in multiple applications like building cooling, renewable energy harvesting, or even dew water production.^2−4^ Surely, building energy efficiency is the most important application of radiative cooling technologies. The growth of human population together with its agglomeration in cities and urban areas is increasing very rapidly the building energy consumption. Currently such consumption accounts for more than 40% of the total energy consumption on the planet,^5^ among which a large portion comes from air conditioning and refrigeration. Indeed, more than half of the electricity demand in big cities of East Asia is for cooling purposes.^6^

Traditionally most of the constructive solutions reported in the state of the art are based on polymer films and coatings^7^ containing certain pigments (typically TiO_2_) that are able to “cool” the building’s surface. Typically, these “cool-roof” paints exhibit modest solar reflectance (*R*_solar_ ∼ 0.85) and good emissivity in the AW (e_AW_ ∼ 0.95), enabling temperature reductions of around 20–30 °C and reasonable energy savings. The advent of radiative cooling materials is expected to take building energy efficiencies to the next level. However, current progress has been largely based on either photonic metamaterials^8,9^ containing scarce and expensive materials with doubtful potential impact in any real building energy solution or hierarchically porous polymer coatings^10,11^ which can be scalable, but at the cost of more expensive industrial manufacturing processes, sacrificing spectral selectivity performance and suffering from durability issues (e.g., ultraviolet UV degradation). Interestingly, more recent works have dug into possible durability solutions concerning environmental aging^12^ or improving the scalability of all-day radiative cooling solutions.^13−15^

In addition to coatings and thin films, certain structural building materials have also been investigated for their radiative cooling properties. Specifically, the cellulose structure of both engineered wood^16^ and biomass fibers^17^ has shown potential for creating bulky structures with daytime radiative cooling capabilities. Despite the fact that concrete and cement-based materials are the most used materials by mankind (just behind water) and play a dominant role in any building and urban design, the potential for exploiting the intrinsic radiative cooling properties of concrete has not yet received the attention it deserves. While a large number of papers have focused on improving the albedo of concrete and pavements,^18,19^ to the best of our knowledge, only three papers have explored the potential of using cement-based materials for daytime radiative cooling purposes. The first one^20^ illustrated that white cements in combination with large amounts of whitening agents (about 150% by weight of cement) turned to give −26.2W/m^2^ as net-radiative cooling power. This value is in stark contrast to the poor cooling power (about −600 W/m^2^) of typical Ordinary Portland Cement (OPC) concretes. In fact, the achieved cooling power is sufficiently close to zero so as to hold promise that cementitious materials can be turned into daytime radiative cooling materials with minor design improvements. The other two published papers (done by some of the authors of this work) explored by computational methods the potential use of concrete for cooling and concomitantly improving the efficiency of solar cells^21^ and the use of dispersive nanoparticles to enable suppressed-scattering windows, allowing for selective thermal emission within a highly reflective sample.^22^

**Figure 1:**
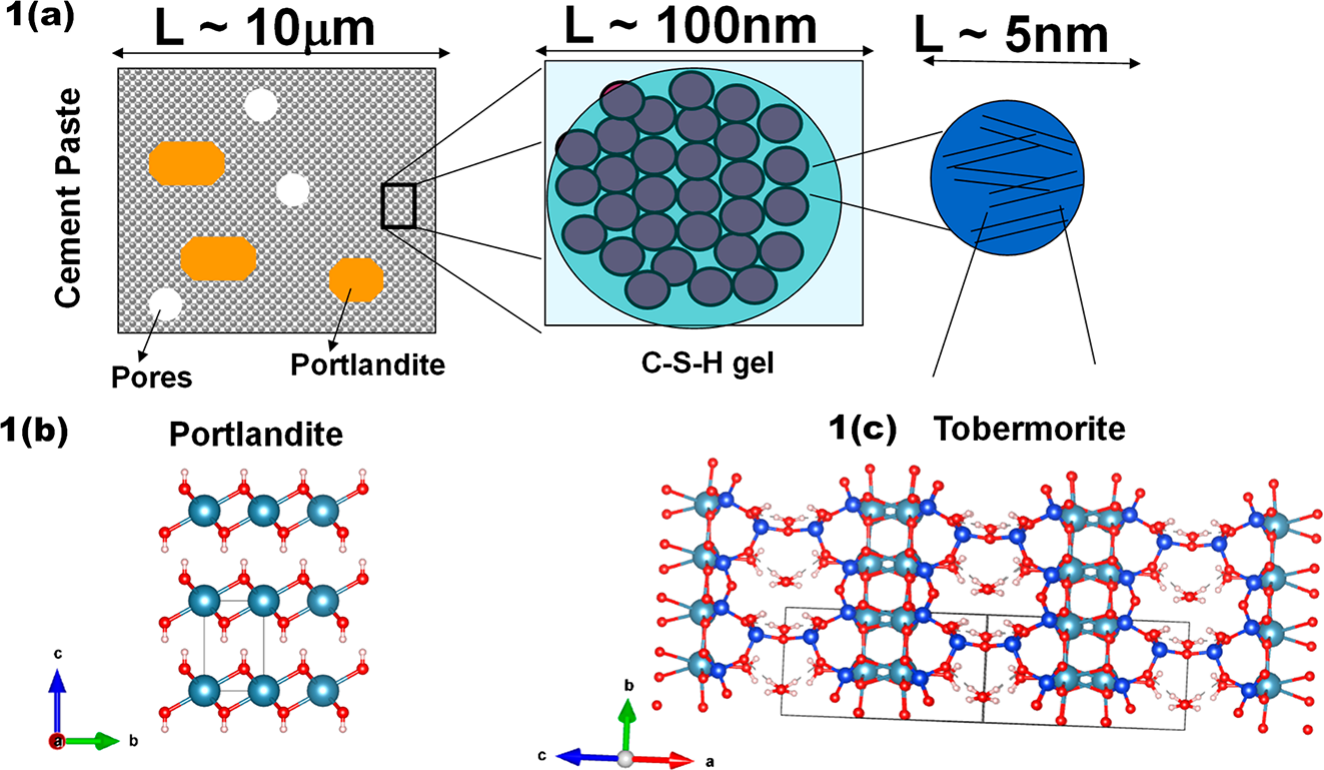
(a) Schematic description of a cement paste. (b) Crystalline structure of Portlandite (Ca(OH)_2_). (c) Crystalline structure of Tobermorite (Ca_5_Si_6_O_16_(OH)_2_·4H_2_O). Notation of the atoms: light blue = Calcium, dark blue = Silicon; red = Oxygen; white = Hydrogen.

A quick insight into concrete’s structure reveals its ample potential for effective optimization. The cementitious matrix is a complex hierarchical porous glassy composite^23,24^ formed upon the reaction of cement grains and water.^25^ In a simplified viewpoint, the cement paste is composed of calcium hydroxide crystallites (Ca(OH)_2_, Portlandite), embedded into an amorphous nanostructured hydration product, the so-called C–S–H gel (see the schematic in Figure 1a).

Portlandite is the mineral name of crystalline calcium hydroxide, with the chemical formula Ca(OH)_2_ or CH in cement chemistry notation. It is the crystalline product present in major quantities in the cement matrix, up to 25% in volume, and appears as massive crystals which can reach sizes of micrometers.^25^ Portlandite has a layered structure, with calcium atoms octahedrally coordinated to OH groups; see Figure 1(b).

Doubtlessly, the most important cement hydration product is the C–S–H gel. It constitutes up to 70% vol of the solid phase and is, therefore, the main component responsible for the material properties. The C–S–H gel is an amorphous and porous material with a variable stoichiometry. The intrinsic structure of the C–S–H gel is still unknown. Although it manifests itself at the scale of a few nanometers in a colloidal fashion,^26−29^ much of the current understanding of the short-range ordering of C–S–H gel has been gained through comparisons with mineral analogs, such as Tobermorite.^30−32^ This calcium silicate hydrate mineral has a sheetlike structure of Ca–O layers ribbed by silicate chains (see Figure 1c).

In this work, the intrinsic radiative cooling properties of Portlandite and Tobermorite minerals are presented, combining reflectance and emissivity measurements with computational simulations. For comparison purposes, the results of both minerals will be discussed in comparison with the values of a real cement paste that has been specifically prepared for this purpose. These results provide valuable insights into the optimization of concrete to achieve effective daytime radiative cooling.

## 2 Experimental Methods

### 2.1 Sample Preparation

Portlandite (Ca(OH)_2_, CH in cement notation) was a >95% pure laboratory reagent purchased from Sigma-Aldrich. In the case of Tobermorite, the used sample corresponds to the Aluminum free Tobermorite sample synthesized in ref (33). Structural and size characterizations of both minerals can be found in the Supporting Information.

For preparing the cement paste, white cement (BL I 52.5 R cement provided by Cementos Cruz (Spain)) was mixed with water in a water-to-cement ratio of 0.3. The cement paste was cast in a cylindrical mold (Ø38 mm × H15 mm) and sealed. After 24 h, the resulting disc was moved to a hermetically closed desiccator with 100% RH and kept at 20 °C. The chemical composition of the white cement (WC) is shown in Table 1.

**Table 1:** WC Composition is Expressed in Terms of Percentages of Oxides^a^

SiO_2_	Al_2_O_3_	Fe_2_O_3_	MnO	MgO	CaO	Na_2_O	K_2_O	TiO_2_	P_2_O_5_	SO_3_	LOI
20.74	3.76	0.16	LD	0.52	62.94	LD	0.64	0.23	0.05	3.26	4.06

a LOI = loss of ignition.

### 2.2 FTIR Experiments

Fourier Transform Infrared (FTIR) experiments were carried out by means of a Jasco 6300 spectrometer in the mid infrared range. Measurements were conducted at ambient temperature (assumed to be 25 °C, unless otherwise stated). In order to minimize errors due to background and lamp intensity variations, background runs were recorded just before every sample measurement.

The vibrational absorption spectra of the samples were recorded in an Attenuated Total Reflectance (ATR) configuration by means of a single reflection diamond ATR from Specac, equipped with a N_2_ purge. Samples were ground to obtain fine powders with a mesh size smaller than 45 μm and directly placed on top of the diamond prism. Pressure was then applied by screwing the anvil until absorption peak intensity reached a maximum value.

The reflectivity of the samples was determined by means of a gold-coated 12° integrating sphere in downward configuration equipped with an MCT detector from PIKE. For these measurements, samples consisted of either solid pieces or fine-grained powders, which were inserted into the sphere according to manufacturer specifications.

### 2.3 Solar Spectral Reflectance Measurements

The sun reflectance of the samples in the range of 0.25 μm–2.5 μm was characterized by using a UV–vis–NIR spectrometer (410-SOLAR). Besides, to correctly calculate the average reflectance over the sun wavelengths (*R*_sun_), we have used the following equation
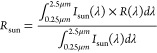
1where the sun irradiation (*I*_sun_) was taken from the ASTM G173 Global Solar spectrum.^34^

## 3 Theoretical Calculations

### 3.1 Materials and Force-Field Methods

All the atomistic simulations have been implemented with the GULP package.^35^ Moreover, the inter(intra) atomic interactions have been described through the nonreactive force field employed in ref (36). This force-field describes the polarizability of the oxygen atoms by a core–shell approach^37^ and allows the estimation of Born effective charges. This is essential for the estimation of the frequency dependent dielectric function (section 3.2).

The starting structures of CH and Tobermorite correspond to the ones experimentally resolved in refs (38) and (39), respectively. The experimental data were later optimized by relaxing the unit cells and atomic forces. The search for the energy local minima followed the Newtons-Raphson procedure with the Broyden-Fletcher-Goldfarb-Shannon (BFGS) protocol^40^ to update the Hessian. The lattice constants obtained after the optimization of Portlandite and Tobermorite structures are listed in Table 2. The models agree well with the experimental values (shown in parentheses). For completeness, the elastic properties were also evaluated. They involve second order derivatives of the energy, and a good description is necessary for the posterior description of the dielectric response. The Bulk (K) and Shear (G) moduli were determined by the Hill definition,^41^ while the Young’s modulus (E) and Poisson’s ratios (ν) were evaluated by assuming isotropic media, i.e. E = (9G) /(3+G/k) and ν = (3–2G/K)/(6 + 2G/K). Like the lattice constants, the utilized force field is recognized for its ability to accurately reproduce the available elastic constants of Portlandite and Tobermorite.^36,42^

**Table 2:** Lattice Constants and Elastic Properties of CH and Tobermorite^a^

	unit cell parameters						elastic properties (GPa)			
	a (Å)	b (Å)	c (Å)	α (deg)	β (deg)	γ (deg)	K	G	E	ν
CH	3.55 (3.59)^38^	3.55 (3.59)	4.94 (4.90)	90 (90)	90 (90)	120 (120)	31.13 (39.6^43^)	13.55 (16.36)^43^	35.50 (40.3,^43^ 36^44^)	0.30
Tobermorite Exp	6.77 (6,735)^39^	7.419 (7.385)	23.222 (22.487)	90 (90)	90 (90)	123.05 (123.25)	72.85 (71 ± 4,^45^ 71 ± 2^46^)	32.98	85.96	0.30

a When available, experimental data is shown in parentheses.

### 3.2 Dielectric Function

The complex dielectric function of cement-based materials can be calculated following the protocol employed in refs (21, 22, and 47). In essence, the underlying idea is that the dielectric function can be calculated in terms of the atomic vibrations (phonons) and more specifically in terms of the oscillator strength Ω as
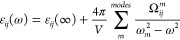
2where the oscillator strength tensor for each vibrational mode *m* depends on the Born effective charges (*q*^B^) and the eigenvector (*e_ij_*) for that mode according to
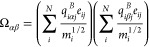
3

To avoid the singularities of eq 2, a small damping term has been used (25 cm^–1^ unless otherwise said). Besides, only the diagonal values of the dielectric function matrix have been considered to estimate the values of the dielectric function; i.e., we have taken ε (ω) ≡ (ε_*xx*_(ω) + ε_*yy*_(ω) + ε_*zz*_(ω))/3 for the real (ε_1_) and imaginary part (ε_2_).

**Figure 2:**
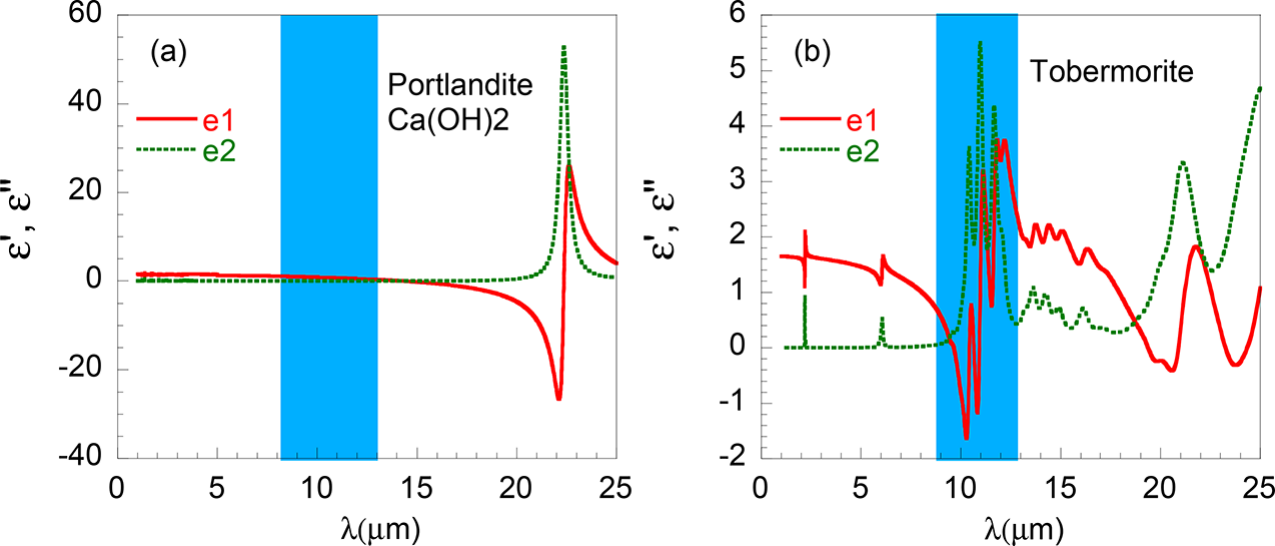
Dielectric functions of Portlandite (a) and Tobermorite (b). In the solid red line is shown the real part, whereas in the dashed green line is the imaginary part.

The dielectric functions obtained for CH and Tobermorite are presented in Figure 2.

From the knowledge of the dielectric function, other measurable properties can be determined like the complex index of refraction
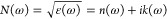
4with refractive index *n*(ω)
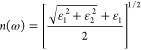
5and extinction coefficient *k*(ω)
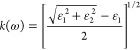
6The absorption coefficient α(ω) and the normal incidence reflectivity *R*(ω) then follow as
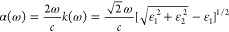
7
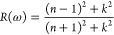
8with *c* denoting the speed of light.

### 3.3 Scattering Efficiencies and Sun Reflectance

Scattering *Q*_sca_ and absorption *Q*_abs_ efficiency factors were obtained via decomposition into spherical harmonics in standard Mie theory^48^
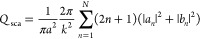
9
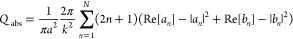
10where *a* is the nanoparticle radius, and *k* = ω/*c* is the propagation constant. The sum runs over the orthogonal contributions of each spherical harmonic and was truncated at *N* = 30 after checking for convergence. *a*_*n*_ and *b*_*n*_ are the *n*^*th*^ order scattering coefficient for TM and TE spherical harmonics, respectively, given by
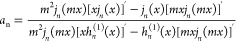
11
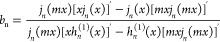
12where we have defined the size parameter *x* = *ka*, and the contrast parameter . *j*_*n*_(*x*) is the spherical Bessel function of the first kind and order *n*, and *h*_*n*_^(1)^(*x*) is the spherical Hankel function of the first kind and order *n*.

### 3.4 Upscaling. Effective Dielectric Functions and Effective Emissivity in the LWIR

To qualitatively interpret the experimental emissivity data in the IR spectral range, we have defined the effective permittivity of white cement (WC) as follows. Starting from the simulated dielectric function of Tobermorite (CSH), we have obtained the effective permittivity ϵ̅_CSH_ of the porous CSH subdomains by solving the following Bruggeman equation^49^
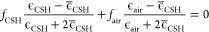
13where *f*_CSH_ is the volume fraction of tobermorite, *f*_air_ is the volume fraction of air, ϵ_CSH_ is the complex permittivity of Tobermorite, and ϵ_air_ is the complex permittivity of air. The introduction of air enables us to account for the porosity when calculating the electromagnetic properties of the sample.

Later, since the C–S–H gel subdomains are mixed with Portlandite (CH) subdomains in the cementitious matrix, we have used the simulated CH dielectric function in a second homogenization step to obtain the white cement (WC) effective permittivity by solving another Bruggeman’s formula
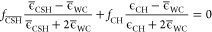
14where the meaning of the symbols is self-explanatory. This equation is valid as long as the pore size is much smaller than the radiation wavelength, which is the case in the IR spectral range, because the pore size of the C–S–H gel belongs to the nanoscale.

This effective-medium approach is similar (although not identical) to the one used in ref (50) to study the terahertz response of cementitious samples.

Finally, in order to describe qualitatively the emission properties of samples made of porous Tobermorite and Portlandite, we have applied the transfer-matrix method (TMM)^51^ to a 1 mm thick planar slab made of these materials, placed onto an ideal bottom reflector, to mimic the placement of the sample on a reflecting sample holder.

By this method, described in detail in ref (51), one can obtain the spectral directional reflectance of the sample *R*_*Ω,λ*_(λ,θ) as a function of the wavelength and incidence angle. The corresponding spectral directional absorbance is *A*_*Ω,λ*_(λ,θ) = 1 – *R*_*Ω,λ*_(λ,θ), since the transmittance is *T*_*Ω,λ*_ = 0. Because of Kirchhoff’s law, the spectral directional emissivity is equal to *A*_*Ω,λ*_(λ,θ).^52^ Finally, one can obtain the spectral absorbance/emissivity *A*_*λ*_(λ) by performing an angular average on the spectral directional absorbance (the same relation can be used to obtain the spectral reflectance):
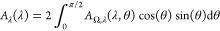
15A more detailed description of the procedure, from effective-medium-theory to transfer-matrix-method simulations, can be found in ref (21).

## 4 Results and Discussion

**Figure 3:**
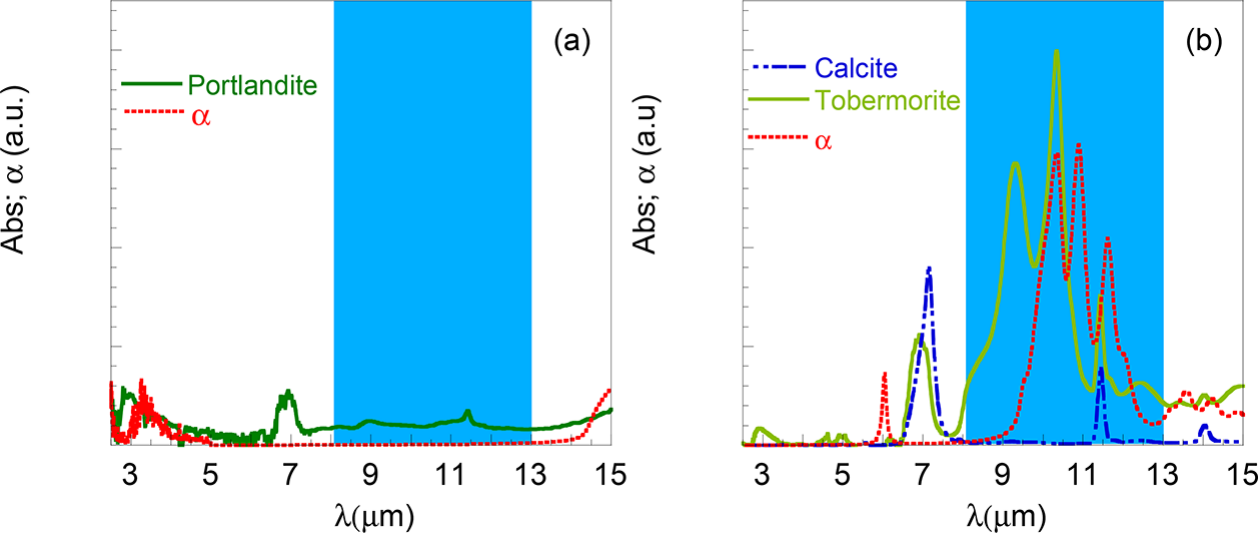
ATR experiments over CH (a) and Tobermorite (b).

Figure 3 displays the ATR FTIR spectra of Portlandite (part a) and Tobermorite (part b) compared to the absorption coefficients obtained by the force-field simulations. The structure of Portlandite is quite simple, and the main absorbance peaks correspond to O–H stretching modes at ∼1.45 μm and Ca–O stretching modes at about 25 μm (outside our experimental observation window). The sample also exhibits a peak at ∼7 μm that indicates the presence of traces of Calcite.

The case of Tobermorite is more complex. As expected, both the experimental observation and computational prediction give prominent absorption peaks within the atmospheric window (shaded blue area), corresponding to Si–O stretching vibrations. Additionally, the experiments present a noticeable peak at 7 μm, which is consistent with the presence (5% BW) of Calcite in the Tobermorite sample. For completeness, the experimental peaks of absorption of Calcite are also plotted in Figure 3(b) (dashed blue line). The extra peaks from the experiments and modeling at 6 and 2.5–3 μm are consequences of water and OH vibrations.

**Figure 4:**
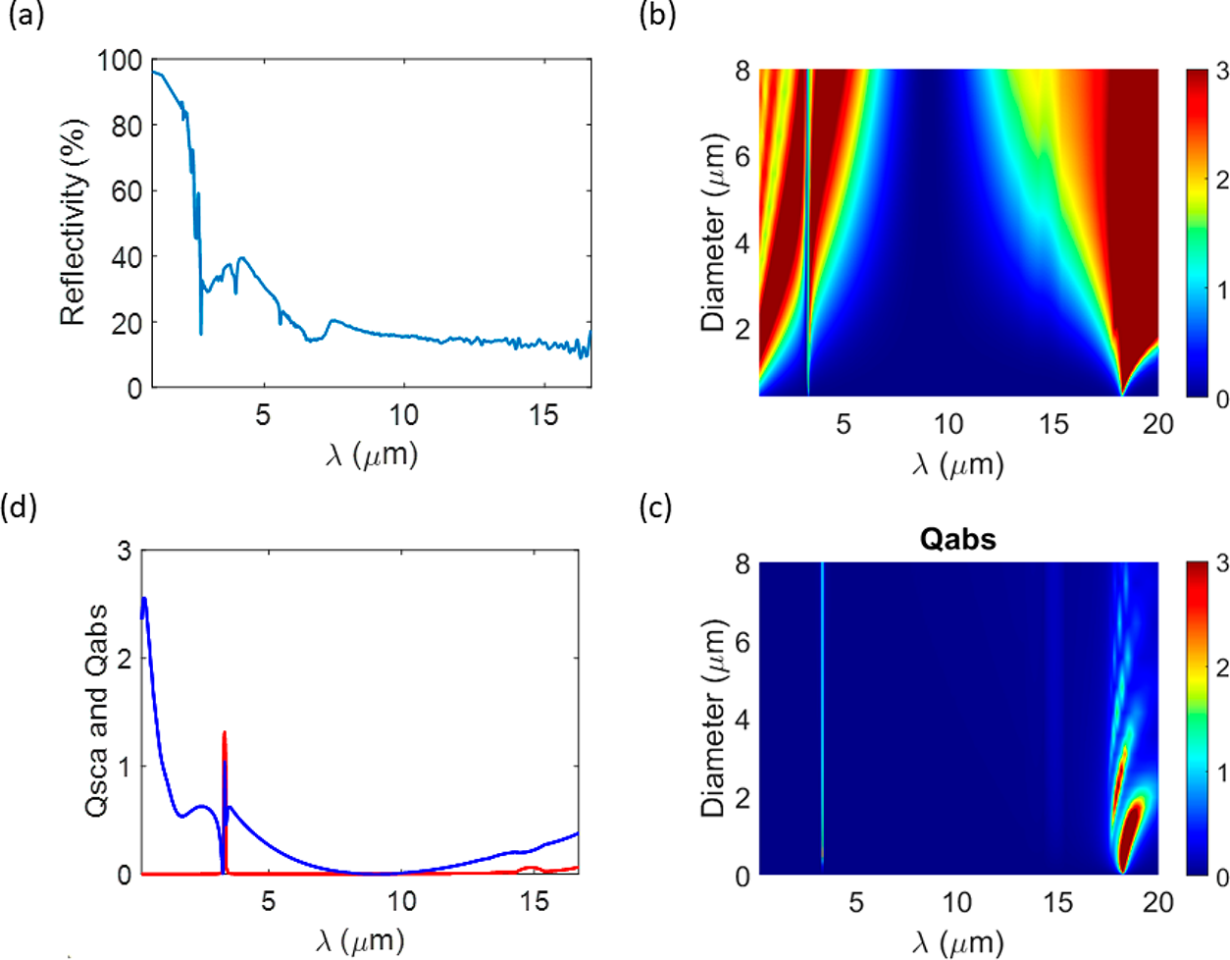
(a) Experimental reflectance of Portlandite (CH). Map of (b) scattering *Q*_*sca*_ and (c) absorption *Q*_*abs*_ efficiency factors as a function of the Portlandite particle size and wavelength. (d) Scattering and absorption efficiency factors for CH after weighting with the experimental size distribution (see the Supporting Information).

Figure 4(a) shows the reflectance of Portlandite. The first thing worth remarking is the high reflectance in the solar domain. The average solar reflectance (*R*_sun_ = 0.93) is clearly higher than those found in concretes with large doses of TiO_2_ (<0.83)^20^ and within the range of threshold values (*R*_sun_ ∼ 0.875–0.95) proposed in ref (10) for achieving subambient cooling. This result indicates that intrinsic cementitious phases such as Portlandite can exhibit daytime radiative cooling properties. Besides, the emissivity of Portlandite within the atmospheric window (e_AW_ = 0.84) turns out to be much larger than the one expected from its weak absorbance in the AW. This is, of course, due to a refractive index close to 1 (note from Figure 2(a) that ε_1_ is close to 1 in the AW).

Figure 4(b),(c) depicts the scattering and absorption efficiency factors of microparticles made of these materials as a function of wavelength and nanoparticle radius, computed by using Mie-scattering theory (see Methods: calculation of the scattering efficiency). The numerical results highlight the critical role of material dispersion on the scattering performance of the composite. At visible frequencies, the permittivity of Portandite approximates the response of a dielectric with negligible dispersion and loss (Figure 2(a)). Therefore, its scattering efficiency (*Q*_scat_) presents multiple Mie-scattering resonances that scale linearly with the nanoparticle size, while featuring a much smaller absorption efficiency *Q*_abs_. This effect justifies the high solar reflectance observed in the experiments. By contrast, a wide “suppressed scattering spectral window”^22^ is observed at longer wavelengths, where the permittivity is close to unity, and the scattering and absorption spectra are devoid of any resonance. At even longer wavelengths, Portlandite presents a strong scattering response in a band in which its permittivity is negative. Consequently, the resonances in such a band have a more “plasmonic-like” character, they are present even for deeply subwavelength sizes, and they are accompanied by larger absorption efficiencies.

Once the scattering and absorption efficiency factors are weighted with the experimental size distribution (Supporting Information), the obtained results can be found in Figure 4(d). The good qualitative agreement between the experimental reflectance and the computed scattering efficiency is remarkable. In fact, the scattering efficiency factor captures well the highest reflectance peaks at 1 μm and the broad one appearing at 4 μm. This result evidences that the response of Portlandite at visible frequencies is largely determined by geometrically driven optical resonances, providing a high sun reflectance, while the response at longer infrared wavelengths is predominantly influenced by material dispersion and loss.

**Figure 5:**
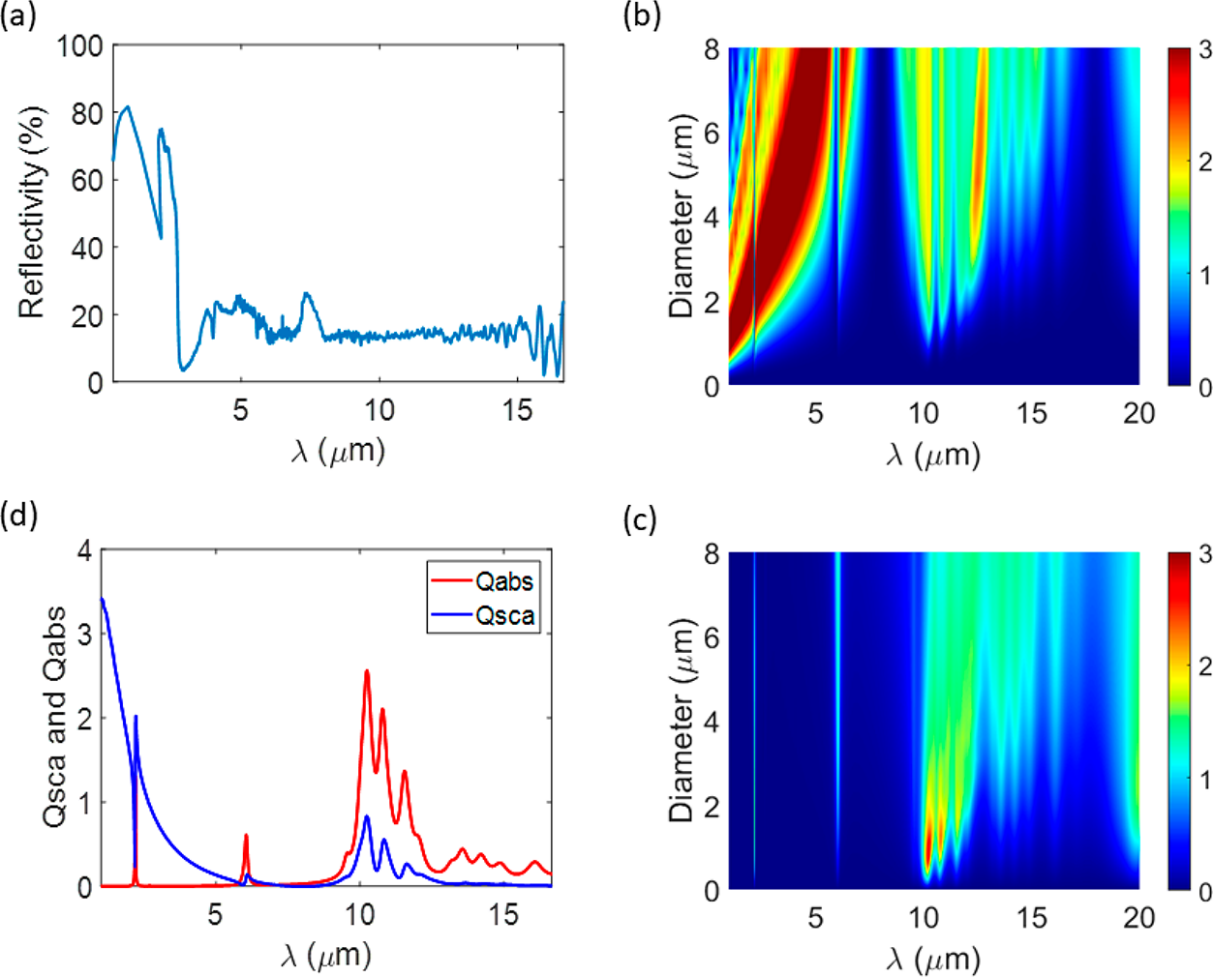
(a) Experimental reflectance of Tobermorite powders. Map of (b) scattering *Q*_*sca*_ and (c) absorption *Q*_*abs*_ efficiency factors as a function of the Tobermorite particle size and wavelength. (d) Scattering and absorption efficiency factors for Tobermorite after weighting with the experimental size distribution (Supporting Information).

Similar conclusions can be drawn for the case of Tobermorite. The experimental reflectance of Tobermorite is displayed in Figure 5(a). Again, the values obtained for the sun reflectance of Tobermorite (*R*_sun_ = 0.75) are higher than those reported for concrete,^20^ though in lower degree. Indeed, the values fall below the aforementioned threshold values required to achieve daytime radiative cooling. Besides, the emissivity of Tobermorite within the atmospheric window (e_AW_ = 0.87) is slightly larger than the one of Portlandite.

Numerical calculations for the absorption and scattering response of Tobermorite are also reported in Figure 5(b),(c), respectively. Similar to Portlandite, the scattering response of Tobermorite in the visible range is characterized by a number of Mie scattering resonances with large *Q*_scat_ and low *Q*_abs_ values, which justify its high sun reflectance. By contrast, the scattering response of Tobermorite at longer infrared wavelengths is characterized by several resonant bands, where both *Q*_scat_ and *Q*_abs_ are significant. Such qualitatively different scattering behavior is a direct consequence of the lossy and dispersive permittivity response of Tobermorite in such a frequency band (see Figure 2b). In turn, the more dominant presence of *Q*_abs_ justifies the lower reflectance.

**Figure 6:**
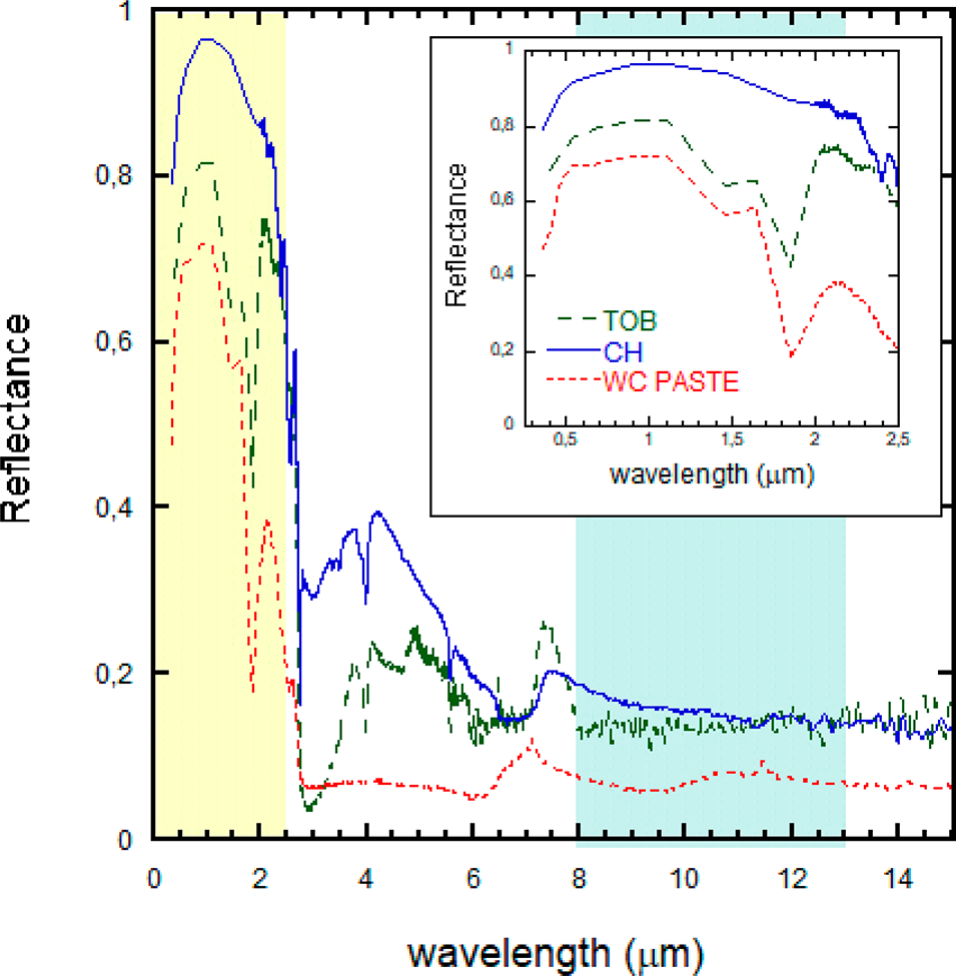
Reflectances of Portlandite (CH) (solid blue line) and Tobermorite (TOB) (dashed green line) in comparison to the one of a white cement paste (WC paste) (dashed red line). The solar domain and atmospheric window are shown by shaded yellow and light bluish green areas, respectively. The solar domain (0.25 μm–2.5 μm) is enlarged in the inset.

After the analysis was done for Portlandite and Tobermorite minerals, it would be interesting to extrapolate our conclusions to realistic cement pastes. Figure 6 illustrates the reflectances measured for Portlandite and Tobermorite in comparison to the one of a realistic 28-day-old white cement paste.

As expected, the solar reflectance of the white cement paste (*R*_sun_ = 0.66) is clearly lower than that of Tobermorite (*R*_sun_ = 0.75) and especially worse than that of Portlandite (*R*_sun_ = 0.93). As previously discussed, the scattering factor analysis has evidenced that this wavelength domain is largely controlled by geometrically driven optical resonances. Therefore, it is clear that tuning the cement composition, as well as its micro and nanostructure, is key to controlling its reflectivity. As a simple rule of the thumb, our study clearly indicates that cementitious composition should be tuned so as to promote the appearance of Portlandite.

The second aspect to discuss is the higher emissivity of the white cement paste (e_AW_ = 0.93) with respect to those of Portlandite (e_AW_ = 0.84) and Tobermorite (e_AW_ = 0.87) within the AW. Moreover, it is worth noting that all three materials, especially the white cement paste, show high and unselective emissivities for wavelengths above 3 μm. These characteristics provide suitable conditions for developing building solutions that utilize nocturnal radiative cooling.^53^ To explore the emissivity of the white cement paste in further detail, we have employed a homogenization scheme (see Methods) to describe a simplified cement paste as shown in Figure 1(a). In fact, three simple models have been considered assuming different volume percentages of Portlandite, Tobermorite, and nanovoids (see Table 3). Essentially C–S–H gel is approximated by Tobermorite with nanovoids.

**Table 3:** Volumetric Fraction of the Three Models of White Cement Paste

	cement paste		CSH gel	
computational models	CH (%)	CSH (%)	Tobermorite (%)	Nanovoid (%)
white cement paste 1 (WC1)	25	75	65	35
white cement paste 2 (WC2)	10	90	65	35
white cement paste 3 (WC3)	25	75	75	25

**Figure 7:**
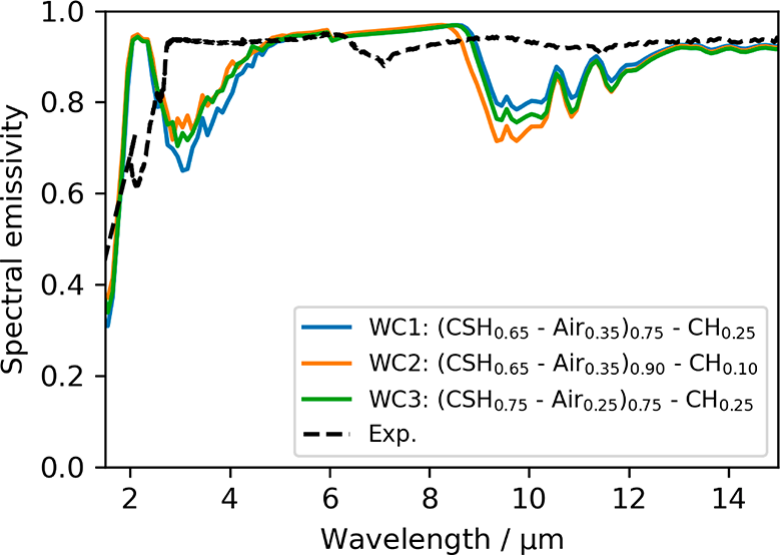
Spectral emissivity of the three models of white cement pastes compared to the experimental data (exp).

The spectral emissivity of the three models is depicted in Figure 7 in comparison to that of the experimental observation. Given the crude approach followed, the models capture reasonably well the overall spectral emissivity of the cement paste.

Beyond the comparison to the experiment, it is clear from the computational models that the WC2 (the one with the lowest CH content) exhibits the lowest emissivity, whereas the WC1 (the one with higher nanoporosity) shows the highest emissivity. These results seem to favor both the presence of Portlandite and the nanoporosity of C–S–H gel to enhance the emissivity of cement pastes in the AW. Keeping in mind that the refractive index of Portlandite is close to the one of air in the AW (see Figure 2(a)), both results stem from the same reason. First, increasing porosity increases the volume fraction having the permittivity of air and reduces the volume fraction having the permittivity of white cement, leading to an effective permittivity gradually closer to the one of air, as can be observed from the refractive index data in Figure S4(a). Second, since the simulated white cement layer is very thick (1 mm) and lossy, all incoming electromagnetic radiation is either immediately reflected at the air/sample interface or absorbed within the sample, i.e. the radiation penetration depth is smaller than the layer thickness. Now, when porosity is increased, the refractive index of the cement becomes closer to the one of air; therefore, the reflectance is reduced, and the absorbance/emissivity is increased. At lower wavelengths, the situation is reversed because the penetration depth is not smaller than the layer thickness anymore due to decreased absorbance. Then, part of the radiation reaches the bottom mirror that reflects it back, crosses again the cement layer, and escapes from the cement/air interface, *de facto* increasing the overall reflectance, as shown in Figure 7 containing the simulated and experimental spectral reflectance data.

## 5 Conclusions

In summary, the intrinsic radiative cooling properties of Portlandite and Tobermorite minerals have been studied by reflectance measurements and computational simulations. In comparison to the values found in normal cement pastes, the solar reflectances of Tobermorite and especially the one of Portlandite are clearly higher. The findings suggest that cement pastes with a higher proportion of Portlandite are more effective at enhancing solar reflectance, which could be achieved through the use of lime-rich binders in practical applications. Additionally, both minerals have intrinsic infrared emissivities lower than those of cement pastes. Our simulations attribute this result to the nanoporosity of the C–S–H gel. Thus, to increase the IR-emittance, cement pastes with the highest possible C–S–H gel nanoporosity should be produced. Appropriate curing conditions involving the temperature and humidity can be adjusted to achieve this.

Overall, the results obtained in this study highlight the potential of using Portlandite and Tobermorite for radiative cooling applications and provide valuable insights into the optimization of concrete composition and porosity to achieve effective daytime and nighttime radiative cooling. Future studies should further explore the potential of these materials as well as other cement-based materials for a range of radiative cooling applications in different climates and environments.

## Supporting Information Available

Characterization of the samples with SEM, XRD, and particle size distributions. Theoretical predictions of the refraction index and extinction coefficients for the three cement paste models (PDF)

## ABBREVIATIONS

AW: atmospheric window
CH: Portlandite
CSH: Tobermorite mineral
C–S–H gel: calcium silicate hydrate gel
WC: white cement

## References

[ref1] Chu S.; Cui Y.; Liu N. The path towards sustainable energy. Nat. Mater. 2017, 16, 16–22. 10.1038/nmat4834. 27994253

[ref2] Zhao D.; Aili A.; Xu S.; Tan G.; Yin X.; Yang R. Radiative sky cooling: Fundamental principles, materials, and applications. Appl. Phys. Rev. 2019, 6, 021306 10.1063/1.5087281.

[ref3] Zhou L.; Rada J.; Tian Y.; Han Y.; Lai Z.; McCabe M. F.; Gan Q. Radiative cooling for energy sustainability: Materials, systems, and applications. Phys. Rev. Materials 2022, 6, 090201 10.1103/PhysRevMaterials.6.090201.

[ref4] Fan S.; Li W. Photonics and thermodynamics concepts in radiative cooling. Nat. Photon 2022, 16, 182–190. 10.1038/s41566-021-00921-9.

[ref5] Pérez-Lombard L.; Ortiz J.; Pout C. A Review on buildings energy consumption information. Energy and Buildings 2008, 40 (3), 394–398. 10.1016/j.enbuild.2007.03.007.

[ref6] Brockett D.; Fridley D.; Lin J. A Tale of Five Cities: The China Residential Energy Consumption Survey; Human and Social Dimensions of Energy Use: Understanding Markets and Demand - 8.29; ACEE Summer Study on Building Energy Efficiency; 2002; pp 8.29–8.40.

[ref7] Mandal J.; Yang Y.; Yu N.; Raman A. P. Future Energy Paints as a Scalable and Effective Radiative Cooling Technology for Buildings. Joule 2020, 4, 1350–1356. 10.1016/j.joule.2020.04.010.

[ref8] Rephaeli E.; Raman A.; Shanhui Fan S. Ultrabroadband Photonic Structures to Achieve High-Performance Daytime Radiative Cooling. Nano Lett. 2013, 13, 1457–1461. 10.1021/nl4004283. 23461597

[ref9] Raman A.; Anoma M.; Zhu L.; Rephaeli E.; Fan S. Passive radiative cooling below ambient air temperature under direct sunlight. Nature 2014, 515, 540–544. 10.1038/nature13883. 25428501

[ref10] Mandal J.; Yanke Fu Y.; Overvig A. C.; Jia M.; Sun K.; Shi N. N.; Zhou H.; Xiao X.; Yu N.; Yang Y. Hierarchically porous polymer coatings for highly efficient passive daytime radiative cooling. Science 2018, 362, 315–319. 10.1126/science.aat9513. 30262632

[ref11] Zhu J.; An Z.; Zhang A.; Du Y.; Zhou X.; Geng Y.; Chen G. Anisotropic porous designed polymer coatings for high-performance passive all-day radiative cooling. iScience 2022, 25, 104126 10.1016/j.isci.2022.104126. 35402873 PMC8983389

[ref12] Song J.; Zhang W.; Sun Z.; Pan M.; Tian F.; Li X.; Ye M.; Deng X. Durable radiative cooling against environmental aging. Nat. Commun. 2022, 13, 4805 10.1038/s41467-022-32409-7. 35973997 PMC9381728

[ref13] Li D.; Liu X.; Li W.; Lin Z.; Zhu B.; Li Z.; Li J.; Li B.; Fan S.; Xie J.; Zhu J. Scalable and hierarchically designed polymer film as a selective thermal emitter for high-performance all-day radiative cooling. Nat. Nanotechnol. 2021, 16, 153–158. 10.1038/s41565-020-00800-4. 33199884

[ref14] Liu R.; Zhou Z.; Mo X.; Liu P.; Duan J.; Zhou J. Green-Manufactured and recyclable coating for subambient daytime radiative cooling. ACS Appl. Mater. Interfaces 2022, 14, 46972–46979. 10.1021/acsami.2c12400. 36215717

[ref15] Zhang Y.; Feng W.-F.; Zhu W.; Shan X.; Lin W.-K.; Guao J.; Li T. Universal color retrofit to polymer-based radiative cooling materials. ACS Appl. Mater. Interfaces 2023, 15 (17), 21008–21015. 10.1021/acsami.3c01324. 37069786

[ref16] Li T.; Zhai Y.; He S.; Wentao Gan; Wei Z.; Heidarinejad M.; Dalgo D.; Mi R.; XZhao X.; Jianwei Song J.; Dai J.; Chen C.; Aili A.; Vellore A.; Martini A.; Yang R.; Srebric J.; Yin X.; Hu L. A radiative cooling structural material. Science 2019, 364, 760–763. 10.1126/science.aau9101. 31123132

[ref17] Chen Y.; Dang B.; Fu J.; Wang C.; Li C.; Sun Q.; Li H. Cellulose-Based Hybrid Structural Material for Radiative Cooling. Nano Lett. 2021, 21 (1), 397–404. 10.1021/acs.nanolett.0c03738. 33301320

[ref18] Levinson R.; Akbari H. Effects of composition and exposure on the solar reflectance of Portland cement concrete. Cem. Concr. Res. 2002, 32, 1679–1698. 10.1016/S0008-8846(02)00835-9.

[ref19] Sanjuán M. Á.; Morales A.; Zaragoza A. Effect of Precast Concrete Pavement Albedo on the Climate Change Mitigation in Spain. Sustainability 2021, 13, 11448 10.3390/su132011448.

[ref20] Lu G.; She W.; Tong X.; Zuo W.; Zhang Y. Radiative cooling potential of cementitious composites: Physical and chemical origins. Cement and Concrete Composites 2021, 119, 104004 10.1016/j.cemconcomp.2021.104004.

[ref21] Cagnoni M.; Tibaldi A.; Dolado J. S.; Cappelluti F. Cementitious materials as promising radiative coolers for solar cells. iScience 2022, 25, 105320 10.1016/j.isci.2022.105320. 36310584 PMC9615327

[ref22] Pérez-Escudero J. M.; Torres-García A. E.; Lezaun C.; Caggiano A.; Peralta I.; Dolado J. S.; Beruete M.; Liberal I. Suppressed-scattering spectral windows for radiative cooling applications. Opt. Express 2023, 31, 6314–6326. 10.1364/OE.477368. 36823891

[ref23] Pellenq R. J.-M.; Kushima A.; Shahsavari R.; Van Vliet K. J.; Buehler M. J.; Yip S.; Ulm F. J. A realistic molecular model of cement hydrates. Proc. Natl. Acad. Sci. U. S. A. 2009, 106 (38), 16102–16107. 10.1073/pnas.0902180106. 19805265 PMC2739865

[ref24] Dolado J. S.; Griebel M.; Hamaekers J.; Heber F. The nano-branched structure of cementitious calcium-silicate-hydrate gel. J. Mater. Chem. 2011, 21, 4445–4449. 10.1039/c0jm04185h.

[ref25] Taylor H. F. W. Cement Chemistry, 2nd ed.; Thomas Telford: London, 1997.

[ref26] Jennings H. M. A model for the microstructure of calcium silicate hydrate in cement paste. Cem. Concr. Res. 2000, 30 (1), 101–116. 10.1016/S0008-8846(99)00209-4.

[ref27] Jennings H. M. Refinements to colloid model of C-S-H in cement: CM-II. Cem. Concr. Res. 2008, 38, 275–289. 10.1016/j.cemconres.2007.10.006.

[ref28] Allen A.; Thomas J.; Jennings H. Composition and density of nanoscale calcium-silicate-hydrate in cement. Nat. Mater. 2007, 6, 311–316. 10.1038/nmat1871. 17384634

[ref29] González-Teresa R.; Dolado J. S.; Ayuela A.; Gimel J.-C. Nanoscale texture development of C-S-H gel: A computational model for nucleation and growth. Appl. Phys. Lett. 2013, 103, 234105 10.1063/1.4838396.

[ref30] Cong X.; Kirkpatrick R. J. 29Si MAS NMR study of the structure of calcium silicate hydrate. Adv. Cem. Based Mater. 1996, 3, 144–156. 10.1016/S1065-7355(96)90046-2.

[ref31] Taylor H. F. Proposed Structure for Calcium Silicate Hydrate Gel. J. Am. Ceram. Soc. 1986, 69, 464–467. 10.1111/j.1151-2916.1986.tb07446.x.

[ref32] Richardson I. G.; Groves G. W. Models for the composition and structure of calcium silicate hydrate (C-S-H) gel in hardened tricalcium silicate pastes. Cem. Concr. Res. 1992, 22, 1001–1010. 10.1016/0008-8846(92)90030-Y.

[ref33] Diez-Garcia M.; Gaitero J. J.; Aguirre F. B.; Erkizia E.; San-Jose J. T.; Aymonier C.; Dolado J. S. Synthesis and Addition of Al-Substituted Tobermorite Particles to Cement Pastes. J. Mater. Civ. Eng. 2022, 34 (12), 04022329 10.1061/(ASCE)MT.1943-5533.0004486.

[ref34] ASTM G173-03 Standard Tables for Reference Solar Spectral Irradiance:Direct Normal and Hemispherical on 37° Tilted surface; 2020.

[ref35] Gale J. D. GULP-a computer program for the symmetry adapted simulation of solids. J. Chem. Soc. Faraday Trans. 1997, 93, 629–637. 10.1039/a606455h.

[ref36] Manzano H.; Dolado J. S.; Ayuela A. Elastic Properties of the Main Species Present in Portland Cement Pastes. Acta Mater. 2009, 57 (5), 1666–1674. 10.1016/j.actamat.2008.12.007.

[ref37] Dick B. G.; Overhauser A. W. Theory of the Dielectric Constants of Alkali Halide. Phys. Rev. 1958, 112, 90–103. 10.1103/PhysRev.112.90.

[ref38] Henderson D. M.; Gutovsky H. S. A nuclear magnetic resonance determination of the hydrogen positions in Ca(OH)_2_ at T = 25 °C. Am. Mineral. 1962, 47, 1231–1251.

[ref39] Merlino S.; Bonaccorsi E.; Armbruster T. Tobermorites: Their real structure and order-disorder (OD) character. Am. Mineral. 1999, 84, 1613–1621. 10.2138/am-1999-1015.

[ref40] Shannon D. F. Conditioning of quasi-Newton methods for function minimization. Math. Comp. 1970, 24, 647–656. 10.1090/S0025-5718-1970-0274029-X.

[ref41] Nye J. F. Physical properties of crystals; Oxford University Press: New York, 1957.

[ref42] Manzano H.; González-Teresa R.; Dolado J. S.; Ayuela A. X-ray spectra and theoretical elastic properties of crystalline calcium silicate hydrates: comparison with cement hydrated gels. Materiales de Construcción 2010, 60 (299), 7–19. 10.3989/mc.2010.57310.

[ref43] Monteiro P. J. M.; Chang C. T. The Elastic Moduli of Calcium Hydroxide. Cem. Concr. Res. 1995, 25, 1605–1609. 10.1016/0008-8846(95)00154-9.

[ref44] Acker P. Micromechanical analysis of creep and shrinkage mechanisms. In Creep, shrinkage and durability of concrete and other quasi-brittle materials; Ulm F.-J., Bazant Z. P., Wittmann F. H., Eds.; Elsevier: Amsterdam, 2001.

[ref45] Dupuis R.; Moon J.; Jeong Y.; Taylor R.; Kang S.-H.; Manzano H.; Ayuela A.; Monteiro P. J. M.; Dolado J. S. Normal and anomalous self-healing mechanism of crystalline calcium silicate hydrates. Cem. Concr. Res. 2021, 142, 106356 10.1016/j.cemconres.2021.106356.

[ref46] Li J.; Zhang W.; Garbev K.; Beuchle G.; Monteiro P. J. M. Influences of crosslinking and Al incorporation on the intrinsic mechanical properties of tobermorite. Cem. Concr. Res. 2020, 136, 106170 10.1016/j.cemconres.2020.106170.

[ref47] Dolado J. S.; Goracci G.; Duque E.; Martauz P.; Zuo Y.; Ye G. THz Fingerprints of Cement-Based Materials. Materials 2020, 13 (18), 4194 10.3390/ma13184194. 32967263 PMC7560472

[ref48] Bohren C. F.; Huffman D. R. Absorption and Scattering of light by Small Particles; A Wiley-Interscience Publication, John Wiley & Sons, Inc.: New York, 1983.

[ref49] Bruggeman D. A. G. Berechnung verschiedener Physikalischer Konstanten von heterogenen Substanzen. I. Dielektrizitätskonstanten und Leitfähigkeiten der Mischkörper aus Isotropen Substanzen. Annalen der Physik 1935, 416 (7), 636–64. 10.1002/andp.19354160705.

[ref50] Guihard V.; Patapy C.; Sanahuja J.; Balayssac J.-P.; Taillade F.; Steck B. Effective Medium Theories in Electromagnetism for the Prediction of Water Content in Cement Pastes. International Journal of Engineering Science 2020, 150, 103273 10.1016/j.ijengsci.2020.103273.

[ref51] Katsidis C. C.; Siapkas D. I. General Transfer-Matrix Method for Optical Multilayer Systems with Coherent, Partially Coherent, and Incoherent Interference. Appl. Opt. 2002, 41 (19), 3978–87. 10.1364/AO.41.003978. 12099609

[ref52] Balaji C. Essentials of Radiation Heat Transfer; Wiley: Ane Books Pvt. Ltd.: Chichester, England; New Delhi, India, 2014.

[ref53] Ahmad M. I.; Jarimi H.; Riffat S. Nocturnal Cooling Technology for Building Applications; Springer Briefs in Applied Sciences and Technology: 2019.

