# Supplementary material for: Radiative Cooling Properties of Portlandite and Tobermorite: Two Cementitious Minerals of Great Relevance in Concrete Science and Technology
Source: ACS Appl Opt Mater. 2023 Jun 23;2(6):1000–9. doi: 10.1021/acsaom.3c00082 (PMC11217932; doi:10.1021/acsaom.3c00082)
Supplement: Supplementary file 1 — ot3c00082_si_001.pdf [file ot3c00082_si_001.pdf]

Supporting Information.

Radiative cooling properties of Portlandite and Tobermorite: two cementitious minerals of great relevance in concrete science and technology

Jorge S. Dolado[*1,2], *Guido Goracci* [1], *Silvia Arrese-Igor* [1], *Andrés Ayuela* [1,2], *Angie Torres* [3,4], *Iñigo Liberal* [3,4], *Miguel Beruete* [3,4], *Juan J. Gaitero* [5], *Matteo Cagnoni* [6], *Federica Cappelluti* [6].

1 Centro de Física de Materiales, CFM (CSIC-UPV/EHU), Paseo Manuel de Lardizabal 5 20170 Donostia/San Sebastian (Spain)

2 Donostia International Physics Center (DIPC), Paseo Manuel de Lardizabal 4 20170 Donostia/San Sebastian (Spain)

3 Department of Electrical, Electronic and Communications Engineering, Public University of Navarre (UPNA), 31006 Pamplona, Spain

4 Institute of Smart Cities (ISC), Public University of Navarre (UPNA), 31006 Pamplona, Spain

5 TECNALIA, Basque Research and Technology Alliance (BRTA), Astondo Bidea, Edificio

700, 48160 Derio.

6 Department of Electronics and Telecommunications, Politecnico di Torino, Corso Duca degli Abruzzi 24, Torino 10129 (Italy)

*** Corresponding author: Jorge S. Dolado E-mail: j.dolado@ehu.eus**

1. Structural and size characterization of Portlandite and Tobermorite (PDF)

Two different scanning electron microscopes (SEM) were used for the study of tobermorite and portlandite samples. In the case of portlandite, the image was acquired using a FEI Quanta 200 ESEM with a Wolfram filament. The sample was deposited on a carbon tape but no coating was use in this case. In the case of Tobermorite, a high-resolution field emission microscope Jeol JSM-7000F with Wolfram filament was used. The sample was deposited on a carbon tape and coated with platinum. SEM images of the Portlandite and Tobermorite samples can be seen in Figure S1.

(a) (b)

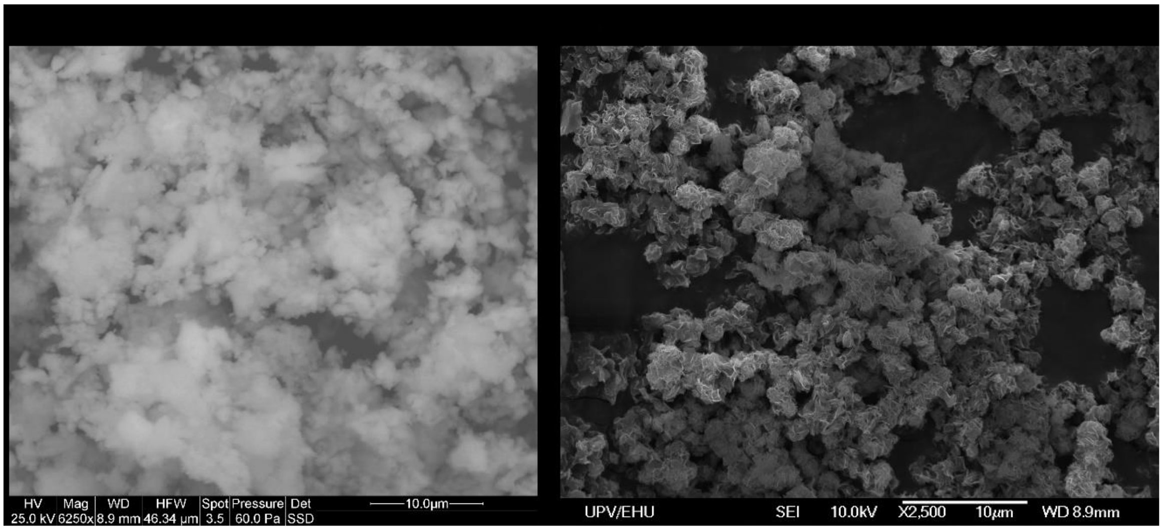

Figure S1. SEM image of (a) Portlandite and (b) Tobermorite.

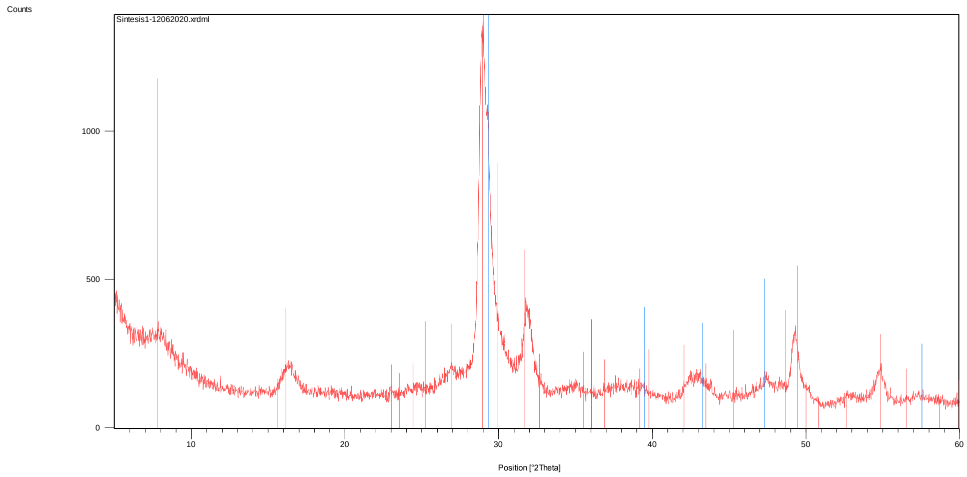

Figure S2. XRD diffractogram of synthesized tobermorite. Red lines correspond to the diffraction peaks of tobermorite 11 (PDF 00-019-1364) and blue lines those of calcite (PDF 00-001-0837) [1].

In the case of Tobermorite, XRD analysis was undertaken (see Figure S2) together with a Rietvelt analysis. XRD measurements were carried out with a Bruker D8 Advance (Billerica, Massachusetts) Series II from 5° to 90° 2θ, with a step size of 0.03°, using Cu K-alpha radiation. Obtained results were processed with DIFFRAC.EVA for phase identification and TOPAS for the quantitative analysis by the Rietveld refining method. For the quantitative analysis, the samples were mixed with 10% of highly crystalline quartz that was used as an internal standard. The results of the RA demonstrate that synthesized material has low crystallinity, being composed by approximately 45% of amorphous material as shown in the Table S1.

Table S1 Mineralogical composition of the synthesized tobermorite obtained by Rietveld analysis.

Sample	Calcite (wt. %)	Tobermorite (wt. %)	Amorphous (wt. %)
Synthetic Tobermorite	5.8	48.4	45.8

Finally, the particle size distribution of the materials was determined using a Malvern Zetasizer Nano instrument, which utilizes dynamic light scattering with a range of 0.3 nm to 15 microns. To prepare the sample, a solution was made by mixing the material with pure water at a ratio of 1:6. The sample was then analysed using the instrument to obtain information on the particle size distribution. The obtained results can be seen in Figure S3.

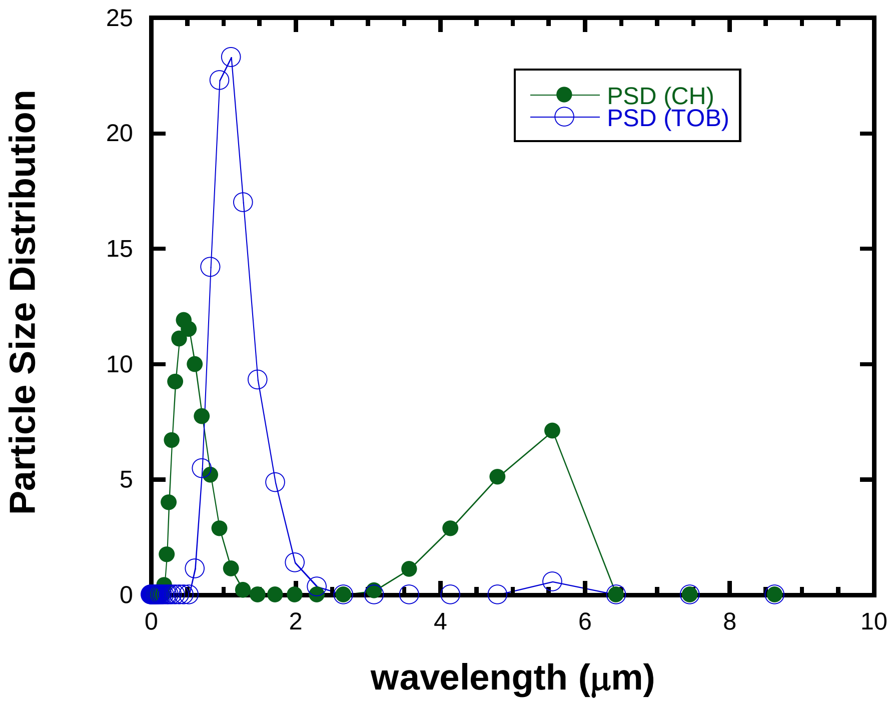

Figure S3. Particle Size Distribution of the Portlandite (CH) and Tobermorite (TOB) particles

2. **Effect of porosity on the homogenization models. Refraction indexes and extinction coefficients.**

The refractive index and extinction coefficients of the simulated White Cement Pastes are shown in Figure S4 (a) and (b) respectively.

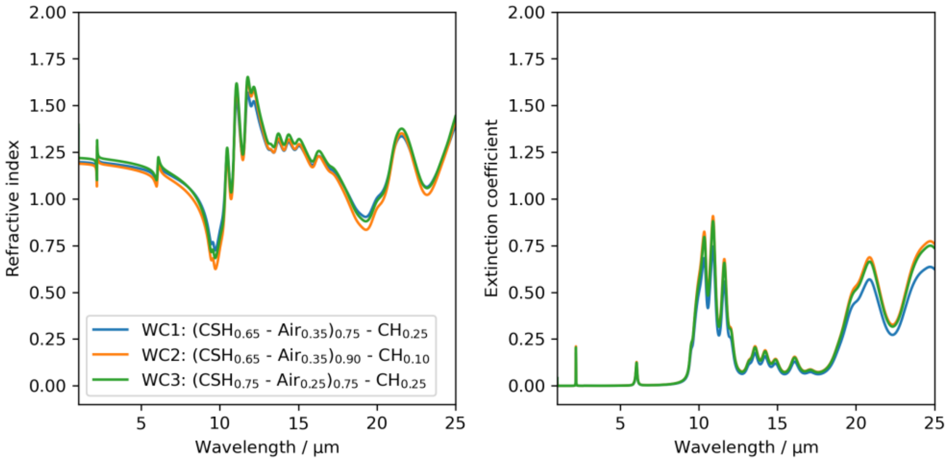

Figure S4. Refractive index (a) and extinction coefficients (b) of the three White Cement (WC) pastes.

References

[1] M. Diez-Garcia, J.J. Gaitero, F.B. Aguirre, E. Erkizia, J.T. San-Jose, C. Aymonier, J.S. Dolado, Synthesis and addition of Al-substituted tobermorite particles to cement pastes, Journal of Materials in Civil Engineering 34 (12), 04022329 (2022), DOI: 10.1061/(ASCE)MT.1943-5533.0004486
